# Analysis of the pathogenic variants of *BRCA1* and *BRCA2* using next-generation sequencing in women with familial breast cancer: a case–control study

**DOI:** 10.1186/s12885-019-5950-4

**Published:** 2019-07-22

**Authors:** Omar Alejandro Zayas-Villanueva, Luis Daniel Campos-Acevedo, José de Jesús Lugo-Trampe, David Hernández-Barajas, Juan Francisco González-Guerrero, María Fernanda Noriega-Iriondo, Ilse Alejandra Ramírez-Sánchez, Laura Elia Martínez-de-Villarreal

**Affiliations:** 1 Department of Genetics, Hospital Universitario “José E. González”, Monterrey, Mexico; 2 Department of Medical Oncology, Hospital Universitario “José E. González”, Monterrey, Mexico

**Keywords:** Pathogenic variant, *BRCA1* and *BRCA2* genes, Triple-negative subtype, Hereditary, Breast cancer

## Abstract

**Background:**

Pathogenic variants (PVs) of BRCA genes entail a lifetime risk of developing breast cancer in 50–85% of carriers. Their prevalence in different populations has been previously reported. However, there is scarce information regarding the most common PVs of these genes in Latin-Americans. This study identified *BRCA1* and *BRCA2* PV frequency in a high-risk female population from Northeastern Mexico and determined the association of these mutations with the patients’ clinical and pathological characteristics.

**Methods:**

Women were divided into three groups: aged ≤ 40 years at diagnosis and/or risk factors for hereditary breast cancer (*n* = 101), aged > 50 years with sporadic breast cancer (*n* = 22), and healthy women (*n* = 72)*.* Their DNA was obtained from peripheral blood samples and the variants were examined by next-generation sequencing with Ion AmpliSeq *BRCA1* and *BRCA2* Panel using next-generation sequencing.

**Results:**

PVs were detected in 13.8% group 1 patients (*BRCA1*, 12 patients; *BRCA2*, 2 patients). Only two patients in group 2 and none in group 3 exhibited *BRCA1* PVs. Variants of uncertain significance were reported in 15.8% patients (*n* = 16). In group 1, patients with the triple-negative subtype, PV frequency was 40% (12/30). Breast cancer prevalence in young women examined in this study was higher than that reported by the National Cancer Institute Surveillance, Epidemiology (15.5% vs. 5.5%, respectively).

**Conclusions:**

The detected *BRCA1* and *BRCA2* PV frequency was similar to that reported in other populations. Our results indicate that clinical data should be evaluated before genetic testing and highly recommend genetic testing in patients with the triple-negative subtype and other clinical aspects.

**Electronic supplementary material:**

The online version of this article (10.1186/s12885-019-5950-4) contains supplementary material, which is available to authorized users.

## Background

Breast cancer is the most common type of cancer among women worldwide and is the main cause of death in developing countries. In 2012, 1.67 million cases were reported worldwide by GLOBOCAN. Hereditary and familial cancers represent approximately 10% of the cases, indicating that 167,000 cases may be attributed to a genetic cause [1].

Approximately 15–40% of hereditary breast cancers occur due to pathogenic variants (PVs) of *BRCA1 (*17q21) and *BRCA2* (13q12–13) [2–5]. *BRCA* PVs may be present in one of eight breast cancer patients aged < 40 years and who have two affected relatives [3]. Carriers of PVs of BRCA genes have a 60% risk of developing breast cancer at the age of 70 years and an 83% risk of developing contralateral breast cancer [6]. Ovarian cancer has high penetrance and association with BRCA PVs. Several other malignancies, such as pancreatic cancer, prostate cancer, and melanoma, have also been associated with mutations in BRCA genes; hence, the patients’ family history should be considered.

The prevalence of *BRCA1/2* germline mutations varies among ethnic groups and geographical zones. Clear variability across Latin American countries has been described, which is explained by the mixture of European, African, and Amerindian ancestors [7]. A founder mutation, ex9-12del, has been described in the Hispanic population from the south of the United States [8], and in an unselected study population from the center of Mexico that was assessed for a family history of cancer and exhibited a mutation frequency of 29% [9]. Mexico is a genetically heterogeneous country, and *BRCA* PV-related information obtained using next-generation sequencing (NGS) is scarce. PVs should be identified for better disease characterization among different populations and for appropriate genetic counseling.

This study established the frequency and type of mutations of *BRCA1* and *BRCA2* in a female population from Northeastern Mexico and determined the correlation of mutations with the patients’ clinical and pathologic characteristics.

## Methods

We performed a case–control study comprising patients from the Centro Universitario Contra el Cáncer at the Hospital Universitario Dr. Jose E. Gonzalez from the Universidad Autónoma de Nuevo León. Subjects (including their parents and grandparents) from Northeastern Mexico (Nuevo León, Tamaulipas, and Coahuila) with high-risk factors for hereditary breast cancer. Enrollment strategy included searching on local data base from January 2005 to August 2015. Women with breast cancer at early age (≤ 40 y) were invited to participate in our study.

Sample size for case-control design considering alpha error 0.05 and beta of 0.8 resulted in 25 persons per group. Despite the calculated sample size, a pre-planned enrollment to recruit 200 people was conducted.

In addition to having a pathological diagnosis of breast cancer, patients were required to meet at least one of the following criteria: age ≤ 40 years at diagnosis [10]; presence of bilateral breast cancer; and three or more relatives with breast cancer, ovarian cancer, pancreatic, prostate, or melanoma cancer; the latter two were independent criteria that did not consider age at diagnosis to be < 40 years.

We include two control groups. Patients with a diagnosis of sporadic breast cancer were termed “positive controls,” and healthy women without a personal or family history of cancer were termed “negative controls.” For the last group, an open invitation was made to medical students and workers for detecting local variants. Inclusion criteria for the healthy group included the following: > 18 y, pedigree with no personal or family history of any cancer, born in the Northeast of Mexico. Informed consent was required for all included patients. Patients meeting the inclusion criteria were selected from the daily hospital outpatient attendance register or from the electronic database of the center and invited to participate by phone. Healthy controls were selected from the general population. An oncologist conducted an interview to obtain the medical history. Clinical data were verified from the electronic medical files of the patients and recorded as baseline data. Peripheral blood sample was taken and analyzed at the molecular laboratory of the genetics department in the university hospital (College of American Pathologists accredited).

### Pathology and mutation analyses

All patients (cases and positive controls) received a diagnosis of invasive breast cancer that was confirmed by anatomopathological analysis at the pathology department of the university hospital. The histologic type of the cancer was determined according to the World Health Organization system [11]. Tumor grade was defined using the Scarff–Bloom–Richardson system. Estrogen and progesterone receptors and HER2 were identified using standard immunohistochemical techniques; hormone receptors were considered positive when at least 1% stain was detected [12]; HER2 was considered positive when “+++” was detected; if “++” was observed, fluorescence in situ hybridization analysis was used for confirmation [13].

DNA extraction was performed using the Qiagen QIAamp DNA Mini Kit, (QIAGEN GmbH, Hilden, Germany), according to the manufacturer’s instructions. Elution was into 100 μL of water.

The entire coding regions of the *BRCA1* and *BRCA2* genes were amplified using the Ion AmpliSeq *BRCA1* and *BRCA2* Panel (Life Technologies, Carlsbad, CA, USA) consisting of three primer pools, covering the target regions in 167 amplicons, including all exons and 10–20 bp of intronic flanking sequences, for both genes. Emulsion polymerase chain reaction (PCR) was performed using the Ion OneTouch™2 Instrument (Cat. No. 4474778), as indicated in Ion PGM™ Template OT2 200 Kit (Publication Number MAN0007221. Rev.A.0; Cat. No. 4480974). Normalized 16-pM sample libraries were pooled and combined with OT2 kit reagents and Ion Sphere particles (ISPs) using an Ion OneTouch ES system (Life Technologies, Carlsbad, CA). Quality control was performed using the Ion Sphere™ Quality Control kit (Life Technologies) to ensure that 10–30% of template-positive ISPs were generated in the emulsion PCR. After the ISP preparation, massively parallel paired-end sequencing was performed with an Ion Torrent Personal Genome Machine (PGM) system using the Ion PGM 200 Sequencing Kit and Ion 316 Chip (Life Technologies), according to the manufacturer’s instructions.

For cases with negative findings, multiple ligation-dependent probe amplification (MLPA) was performed to search for large genomic alterations, duplications, or deletions of one or more exons, as per guideline recommendations [14]. The SALSA MLPA P087-C1 BRCA1 and SALSA MLPA P077-A3 BRCA2 test kits (MRC-Holland, Amsterdam, Netherlands) were used in accordance with the manufacturer’s instructions.

### Data analysis

The raw data were analyzed using torrent suite software v5.0.4 (Life technologies). Coverage analysis was performed using the coverage analysis plug-in v5.0.2.0. Mutations were detected using the Variant Caller plug-in v5.0.2.1 (Life Technologies). To eliminate erroneous base calling, two filtering steps were used to generate final variant calling. The first filter was set at an average total coverage depth of > 80, each variant coverage of > 20, a variant frequency of each sample of > 5, and *p*-value of < 0.01. The second filter was employed by visually examining mutations using Integrative Genomics Viewer software (https://software.broadinstitute.org/software/igv/). Ion Reporter 5.0 was used for variant annotation and classification.

After the filtrations, all variants identified through NGS (silent, missense, nonsense, frameshift, and splicing variants) were compared with variants in the 1000 Genomes Project (http://www.1000genomes.org/) for different ethnic populations, using ExAC (http://exac.broadinstitute.org/about) and 72 in-house controls. All mutations were also checked against the UMD, LOVD, kConFab, HGMD, and ClinVar databases, and were regarded as “pathogenic” if classified as such in these databases. The missense variants were annotated using the wANNOVAR web site (http://wannovar.wglab.org), which provides tools such as SIFT, PolyPhen-II HDIV, PolyPhen-II HVAR, LRT, Mutation Taster, Mutation Assessor, FATHMM, PROVEAN, VEST3, MetaLR and M-CAP to predict the effect of amino acid substitution for each missense mutation. Every missense mutation was scored as damaging or benign using the 11 prediction tools. If the missense mutation was scored as damaging by five or more of the prediction tools, the mutation was classified as a “damaging” mutation, and if it was scored by less than three, the mutation was classified as “benign”. The detected variants are classified based on the criteria of the ENIGMA (Evidence-based Network for the Interpretation of Germline Mutant Alleles) consortium (https://enigmaconsortium.org) and described as recommended by Human Genome Variation Society (https://www.hgvs.org/) using as RefSeq: NM_007294.3 and NM_000059.3. To verify if the PVs identified were true variants or sequencing artifacts, point mutations classified as PV were confirmed by Sanger sequencing, using the BigDye Terminator v3.1 sequencing kit and the ABI PRISM 3130 Genetic Analyzer (Life Technologies).

### Statistical analysis

Patient characteristics were tabulated, and description data are presented as the mean with standard deviations and proportions. Comparisons between groups (familial hereditary vs. sporadic and carriers vs. noncarriers) were performed using a t-test for two independent means and chi-squared test for two proportions expressed as percentages. Odds ratios (ORs) were calculated for age, bilateral cancer, family history, and triple-negative variables. SPSS version 20 (IBM, Armonk, NY) for Windows 7 was used for statistical analysis.

## Results

All subjects were born in Northeastern Mexico. From January 2005 to August 2015, 3,065 patients were registered in the hospital database. We eliminated 265 patients because the reported age was not reliable. There were 436 patients (15.5%) aged ≤ 40 years at diagnosis, among whom 335 were either not located or did not agree to participate. 101 patients were included with early age breast cancer and/or familial/hereditary breast cancer, 22 patients with sporadic cancer (positive controls), and 72 healthy women (negative controls). The clinical characteristics of the patients and positive control groups are shown in Table 1. As expected, the mean age of the familial breast cancer group was significantly lower (36.9 ± 5.2 years). No statistically significant differences were noted between the groups. Regarding tumor histopathology, 53% of patients in the hereditary cancer group exhibited nuclear grade 3 compared with only 10% in the sporadic cancer group (*p* < 0.001).

**Table 1:** Baseline characteristics of groups 1 & 2; risk factors, tumor characteristics, and treatment

Characteristic	Group 1	Group 2
	*n* = 101	*n* = 22
Age at diagnosis,		
(years); ±SD	36.9 ± 5.2	53.1 ± 4.8
Familial cancer		
n (%)	54 (53%)	0
BMI,		
mean; ±SD	27.7 ± 5.3	27.1 ± 4.2
Age at menarche		
mean ± SD	12.3 ± 1.4	12.7 ± 1.3
Parity		
mean ± SD	2.4 ± 1.6	3.6 ± 2.1
Nuliparous; n (%)	18 (18%)	1 (5%)
Age at first pregnancy		
mean ± SD	21 ± 5.4	23 ± 5.6
Breastfeeding; n (%)	48/76 (63%)	11/20 (55%)
Contraceptive use; n (%)	38/97 (39%)	8/20 (40%)
Smoking; n (%)	17 (17%)	7 (32%)
Histology; n (%)		
Ductal	80/90 (89%)	18/22 (82%)
Other	10/90 (11%)	4/22 (18%)
Nuclear Grade; n (%)		
2	31/80 (39%)	18/21 (86%)
3	42/80 (53%)	2/21 (10%)
Stage; n (%)		
0	1/101 (1%)	1. (0)
I–II	57/101 (56%)	17/22 (77)
III	39/101 (39%)	5/22 (23)
IV	4/101 (4%)	0 (0)
T; n (%)		
0–1	29/100 (29%)	11/22 (50)
2	37/100 (37%)	7/22 (32)
3	25/100 (25%)	3/22 (14)
4	9/100 (9%)	1/22 (4)
N; n (%)		
Positive	59/99 (60%)	12/22 (55%)
IHC; n (%)		
ER (+)	52/95 (55%)	22/22 (100%)
PR (+)	47/95 (49%)	21/22 (95%)
HER (+)	19/93 (20%)	5/22 (23%)
Triple negative	30/93 (32%)	0 (0%)
Surgery type; n (%)		
Radical mastectomy	80/99 (81%)	10/22 (45%)
Breast conservative	18/99 (18%)	12/22 (55%)
Chemotherapy; n (%)		
Neoadjuvant	28/100 (28%)	4/22 (18%)
Adjuvant	62/100 (62%)	15/22 (68%)
Anthrac. based	9/62 (15%)	7/15 (40%)
Anthrac/Taxane.	45/62 (72%)	6/15 (47%)

*SD* standard deviation, *IHC* immunohistochemical analysis, *ER* estrogen receptor, *PR* progesterone receptor; all means, and proportions were estimated for all the patients in each group, unless otherwise specified in the table

PGM sequencing of these 195 patients had an average of 60,463 reads per patients, with the mean read length being 113 bp. The average read depth per sample was 330X, with the mean percentage of reads on target being 92% and uniformity of base coverage being 96.3%. PV analysis of *BRCA1* and *BRCA2* revealed 16 mutation carriers. 14 carriers (13.8%) present 10 different PVs in group 1 (Table 2). Overall, 12 different PVs were detected, and most of them (82%) were of *BRCA1* (13/16), whereas only 18% (3/17) were of *BRCA2*. Among these, 11 variants were classified as pathogenic and one as likely pathogenic. Sixteen variants were identified, eight (50%) through NGS, and eight (50%) using MLPA. PVs identified with NGS were re-sequenced by Sanger and all were true variants for a validation rate of 100%. Two deletions, ex9-12del and ex16-17del accounted for 42.8% among carriers in the familial-hereditary group, 21.4% (3/14) respectively. Two PV’s (1 in *BRCA1* and one in *BRCA2*) were detected in the positive control group. No PV’s were detected in the 72 healthy women. Results of total variants are summarized in are reported in Additional file 1: Table S1.

**Table 2:** Pathogenic variants detected by BRCA Panel

Gene	Transcript	Locus	Exon	Intron	Frequency	Quality	Coding	Protein	Function	dbSNP	Clinical Significance	Enigma Classification	No. of Patients
*BRCA1*	NM_007294.3	chr17:41245972	10	–	47.1	1980.35	c.1576C > T	p.Gln526Ter	Nonsense	rs80356984	Pathogenic	C 5	1
*BRCA1*	NM_007294.3	chr17:41234451	12	–	47	410.40	c.4327C > T	p.Arg1443Ter	Nonsense	rs41293455	Pathogenic	C 5	1
*BRCA1*	NM_007294.3	chr17:41215920	17	–	50	533.95	c.5123C > A	p.Ala1708Glu	Missense	rs28897696	Pathogenic	C 5	1
*BRCA1*	NM_007294.3	chr17:41201198	21	–	43.2	387.66	c.5346G > A	p.Trp1782Ter	Nonsense	rs80357284	Pathogenic	C 5	1
*BRCA1*	NM_007294.3	chr17:41246724	9	–	43.4	242.36	c.682_683insAGCCATGTGG	p.Gly228Glufs*15	Frameshift	–	Pathogenic	C 5	1
*BRCA1*	NM_007294.3	chr17:41197819–41203079	21–22	–	–	–	**c.5278-?_5406 +?dup	p.?	Duplication	–	Pathogenic	C5	1
*BRCA1*	NM_007294.3	chr17:41215390–41222944	16–17	–	–	–	**c.4676-?_5074 +?del	p.?	Deletion	–	Pathogenic	C5	3
*BRCA1*	NM_007294.3	chr17:41228631–41249260	9–12	–	–	–	** c.548-?_4185 +?del	p.?	Deletion	–	Pathogenic	C5	3
*BRCA1*	NM_007294.3	chr17:41267796–41278345	P-2	–	–	–	**c.-716-?_80 +?del	p.?	Deletion	–	Pathogenic	C5	1
*BRCA2*	NM_000059.3	chr13:32945092	–	19	38.5	1538.41	c.8488-1G > A	p.?	Splice Acceptor Site	rs397507404	Pathogenic	C5	1
*BRCA2*	NM_000059.3	chr13:32954050	23	–	81.8	450.72	c.9117G > A	p.Pro3039=	synonymous	rs28897756	Pathogenic	C5	1
*BRCA2*	NM_000059.3	chr13:32944584	12	–	49.6	503.98	*c.8377G > A	p.Gly2793Arg	Missense	rs80359082	Pathogenic	C5	1

Abbreviations: *dbSNP* Single Nucleotide Polymorphisms database, *ENIGMA* Evidence-based Network for the Interpretation of Germline Mutant Alleles, *C5* Class 5 – (there is significant evidence to suggest that this variant is a dominant high-risk pathogenic variant

*Pathogenic variants on control group

**Detected by MLPA method

A comparison of demographic and clinical characteristics between the mutation and non-mutation groups only revealed a difference in the frequency of breast feeding (35.2% of mutated patients performed breastfeeding compared with 59.3% of non-mutated patients; *p* = 0.04). Regarding tumor characteristics, the triple-negative subtype was more frequently observed in patients with *BRCA* PVs than in those without PVs (65% vs. 22.6%; *p* < 0.001). The association of the triple-negative subtype with PVs of *BRCA* exhibited an OR of 6.4 (95% CI, 2.22–18.70). Other clinical and tumor characteristics did not statistically differ between the mutation and non-mutation groups (Table 3).

**Table 3:** Hereditary demographics, risk factors, and tumor characteristics

Variable	*BRCA* mutation carriers *n* = 21	Non-carriers *n* = 80	
Age: m (SD)	35.6 (4.5)	37.3 (5.4)	*p* = 0.18
Family history of cancer	66.6%	50.0%	*p* = 0.17
Body mass index, m (SD)	27.8 (5.0)	27.7 (5.5)	*p* = 0.94
Age at menarche m (SD)	12.1 (2.9)	12.1 (1.7)	*p* = 1.00
No. pregnancy m (SD)	2.4 (2.1)	2.3 (1.5)	*p* = 0.80
Nulligravid (%)	19.0%	17.7%	*p* = 0.89
Age at first birth, m (SD)	20.7 (4.9)	21.6 (5.6)	*p* = 0.50
Breastfeeding %	35.2%	59.3%	*p* = 0.04
Contraception (%)	40%	37.9%	*p* = 0.86
Smoking (%)	14.2%	17.5%	*p* = 0.72
Histology, ductal. (%)	88%	89%	*p* = 0.90
Nuclear grade. (%)			
2	27%	41.5%	*p* = 0.28
3	67%	49.2%	*p* = 0.22
Stage. (%)			
0	4.7%	0%	*p* = 0.0002
I-II	71.4%	52.5%	*p* = 0.12
III	23.8%	42.5%	*p* = 0.11
IV	0%	5%	*p* = 0.29
T			
0–1	19%	31.6%	*p* = 0.26
2	57%	31.6%	*p* = 0.03
3	19%	26.6%	*p* = 0.47
4	4.7%	10.2%	*p* = 0.43
N Positive	50%	62%	*p* = 0.33
IHC			
ER (+)	30%	60.5%	*p* = 0.01
PR (+)	30%	53.9%	*p* = 0.058
HER (+)	16%	21.6%	*p* = 0.57
Triple Negative.	65%	22.6%	*p* = < 0.001

*m* mean, *SD* standard deviation, *IHC* immunohistochemical analysis, *ER* estrogen receptor, *PR* progesterone receptor

## Discussion

The university oncology center serves the northeast region of Mexico. At least 30% come from other states and they are mostly low-income individuals who live in rural areas. So, the need for phone contact for participation and travel-related costs provoke low rates of participation, compared with the population found in the local database; however, the sample size was complete, as previously estimated.

Due to the lack of genetic characterization of BRCA genes in Mexico, 72 healthy women were included as control negative. Most of the previous studies are on Hispanics from diverse origins [7, 8]. There is scarce information in Mexico for healthy population. Local variants were not detected among healthy controls. Less information exists in Mexico about BRCA variants in this population.

The frequency of PVs in BRCA1/2 genes reported by clinics that attend to high-genetic-risk populations in North America is approximately 9.3% [15]; By contrast, the frequency of PVs reported in the Hispanic population from Southwestern United States is as high as 25% [16]. In the present case–control study, a frequency of 13.8% of PVs was observed in a population from Northeastern Mexico, which is like that previously reported [17]. In Mexico, the frequency of PVs of these genes has been reported to be from 4 to 27%, depending on the studied population (for example, cases with risk factors and sporadic cases) and tumor characteristics [17–20]. Particularly, among populations with familial/hereditary characteristics, the frequency was 10.2% in Mexico, which is not statistically different from our study (*p* = 0.14) [17].

Over 1,500 clinically significant PVs have been described for each BRCA gene [21, 22]. Among studies published in Mexican population 53 pathogenic genomic variants of BRCA1/2 (24 in patients with early onset or a family history of breast cancer, 28 in unselected populations, and one in both unselected populations) have been reported. Only one PV, a large genomic rearrangement (c.548-?_4185 +?del), which is considered a founder mutation in Mexicans, was recurrent in different studies [9, 20]. Torres-Mejía et al. and Villarreal et al. Reported the frequency of this PV was 1% or 22% among carriers and 9.4% or 42% among carriers, respectively. In our study we detected this PV in 2.9% or 21.4% among group 1 carriers%. Inclusion criteria among these studies are different, going from an unselected population, triple negative in patients younger than 50 y and in this study in an early breast cancer and/or family history. This data must be noticed because of the high spectrum of PVs in our population.

We discovered one PV, predicted to be deleterious, not previously reported; c.682_683insAGCCATGTGG; p.Gly228Glufs*15. This last PV was detected in an early age onset breast cancer patient, 33 y at the time of diagnosis, with bilateral cancer and triple negative subtype. Two variants, p.Ser186Tyr and p.Thr1561Ile, are currently classified in several databases as benign. These two patients had early onset breast cancer at the age of 39 y with HER overexpression and 37 y with luminal subtype, none had family history. Nevertheless, according to the pathogenic predictors used in this study and considering the low frequency of these variants reported in 1000 Genomes Project, gnomAD, ExAc, we suggest further research for proper classification.

Some laboratories have been introducing multiplex assays, which analyze the most common genetic variants. In the present study, in addition to the founder genomic variant, all patients analyzed up to date were carriers of different PVs. From these data, we can infer that the use of these panels may provide missing information at least for Mexican populations.

New technologies such as NGS are currently being used for gene testing because they save time, are cost-effective, and have a higher sensitivity and specificity [23]. Nevertheless, it is important to use at least two different genomic technologies to rule out genomic variants, because as observed in this study, the use of MLPA enabled the identification of 38% of the PVs.

Because these technologies are not available in all clinical settings, clinical criteria should be considered to select patients for genetic testing. Recently, the criteria for hereditary breast cancer has been changing, with an expansion in the risk-related age range, family history, and pathologic characteristics [23] Patients with the triple-negative phenotype may even be of older age (> 50 years) [24]. In a previous study in Australia and Poland comprising patients unselected by age or family history of cancer, the prevalence was between 9.3 and 9.9% [25]. In a similar study of a Mexican population with a median age of 43 years (range, 23–50 years) and the triple-negative phenotype, the prevalence of PVs of *BRCA* was 23% [26]. In this study, the frequency was as high as 43.3%, representing 65% (OR, 6.4; 95% CI, 2.2–18.7) of the patients with PVs, as mentioned previously. This finding indicates the importance of clinical aspects in decision making with regards to the need for gene testing.

Personal and environmental data are important characteristics for counseling and for decreasing the risk of breast cancer and other malignancies to some extent. Breastfeeding is considered an important protective factor for cancer development. In the present study, less proportion of women with PVs performed breastfeeding. Accordingly, it is important to recommend breastfeeding to carriers of *BRCA* PVs. This last modifiable risk factor has been described to be significant in decreasing the risk for breast cancer, with a relative risk of 0.63 (95% CI, 0.46–0.86) in mutated *BRCA1* populations [27]. To the best of our knowledge, this is the first study to compare the effect of breastfeeding on breast cancer of the young between carriers of *BRCA* PVs and noncarriers in Mexico.

*BRCA* gene status is important for the selection of treatment. The use of platinum analogs has shown more benefits in metastasis cases, with a favorable response of 54% compared with 19% for the use of other therapies [28]. Novel therapies that involve poly (ADP-ribose) polymerase inhibitors have shown advantages when used in combination with chemotherapy for BRCA-positive cases [29]. This highlights the need of gene testing not only for genetic counseling but also for treatment. In this study, therapy was not decided on the basis of the BRCA gene status.

## Conclusions

In the present study, *BRCA* PVs were detected with a frequency of 20% in a high-risk population, using Ion AmpliSeq *BRCA1* and *BRCA2* Panel together with MLPA. Because there is a high variability in the type and frequency of BRCA gene variants in the Mexican population, we propose the use of these technologies. We also state that clinical aspects can facilitate decision making regarding the need for *BRCA* analysis. The triple-negative subtype has a good correlation with *BRCA* mutations, so it is difficult to exclude this population from analysis. Strategies to promote a healthier environment must be included in the medical advice to patients. Breastfeeding as a modifiable risk factor should be part of the analyses in future studies to determine the impact in high-risk groups of not only breast cancer, but also ovarian cancer.

## Additional file

Additional file 1: **Table S1.** Genetic Database. Excel file with total data about genetic variants in BRCA1/2 about the three groups; Healthy, Sporadic, and Hereditary. (XLSX 83 kb)

## Data Availability

The datasets used by the authors are available on reasonable request to the corresponding author at laelmar@yahoo.com.mx.

## Abbreviations

ISPs: Ion Sphere Particles
MLPA: Multiple Ligation-dependent probe amplification
NGS: Next Generation Sequencing
ORs: Odds Ratios
PCR: Polymerase Chain Reaction
PV: Pathogenic Variant
